# Heart Team risk assessment with angiography‐derived fractional flow reserve determining the optimal revascularization strategy in patients with multivessel disease: Trial design and rationale for the DECISION QFR randomized trial

**DOI:** 10.1002/clc.23821

**Published:** 2022-03-31

**Authors:** Kotaro Miyata, Taku Asano, Akira Saito, Kohei Abe, Toru Tanigaki, Masahiro Hoshino, Tomoaki Kobayashi, Yoshimitsu Takaoka, Takayoshi Kanie, Manabu Yamasaki, Kunihiko Yoshino, Naoki Wakabayashi, Koki Ouchi, Hiroyuki Kodama, Yumi Shiina, Rihito Tamaki, Yosuke Nishihata, Keita Masuda, Takahiro Suzuki, Hideaki Nonaka, Hiroki Emori, Yuki Katagiri, Yosuke Miyazaki, Yohei Sotomi, Motoki Yasunaga, Norihiro Kogame, Shoichi Kuramitsu, Johan H. C. Reiber, Takayuki Okamura, Yoshiharu Higuchi, Tsunekazu Kakuta, Hiroyasu Misumi, Nobuyuki Komiyama, Hitoshi Matsuo, Kengo Tanabe, DECISION QFR investigators

**Affiliations:** ^1^ Department of Cardiovascular Medicine, St. Luke's International Hospital St. Luke's International University Tokyo Japan; ^2^ Department of Cardiovascular Surgery, St. Luke's International Hospital St. Luke's International University Tokyo Japan; ^3^ Department of Cardiovascular Medicine Gifu Heart Center Gifu Japan; ^4^ Division of Cardiovascular Medicine Tsuchiura Kyodo General Hospital Ibaraki Japan; ^5^ Department of Cardiology Osaka Police Hospital Osaka Japan; ^6^ Department of Radiology, St. Luke's International Hospital St. Luke's International University Tokyo Japan; ^7^ Division of Cardiology Mitsui Memorial Hospital Tokyo Japan; ^8^ Department of Cardiovascular Medicine Wakayama Medical University Wakayama Japan; ^9^ Department of Cardiovascular Medicine Sapporo Higashi Tokushukai Hospital Sapporo Japan; ^10^ Division of Cardiology, Department of Medicine and Clinical Science Yamaguchi University Graduate School of Medicine Yamaguchi Japan; ^11^ Department of Cardiovascular Medicine Osaka University Graduate School of Medicine Osaka Japan; ^12^ Division of Cardiovascular Medicine Toho University Ohashi Medical Center Meguro Tokyo Japan; ^13^ Department of Cardiology Kokura Memorial Hospital Kitakyushu Japan; ^14^ Department of Radiology Leiden University Medical Center Leiden the Netherlands

**Keywords:** decision‐making, functional SYNTAX score, Heart Team, quantitative flow ratio, SYNTAX score II 2020

## Abstract

In patients with multivessel disease (MVD), functional information on lesions improves the prognostic capability of the SYNTAX score. Quantitative flow ratio (QFR®) is an angiography‐derived fractional flow reserve (FFR) that does not require a pressure wire or pharmacological hyperemia. We aimed to investigate the feasibility of QFR‐based patient information in Heart Teams' discussions to determine the optimal revascularization strategy for patients with MVD. We hypothesized that there is an acceptable agreement between treatment recommendations based on the QFR approach and recommendation based on the FFR approach. The DECISION QFR study is a prospective, multicenter, randomized controlled trial that will include patients with MVD who require revascularization. Two Heart Teams comprising cardiologists and cardiac surgeons will be randomized to select a revascularization strategy (percutaneous coronary intervention or coronary artery bypass graft) according to patient information either based on QFR or on FFR. All 260 patients will be assessed by both teams with reference to the anatomical and functional SYNTAX score/SYNTAX score II 2020 derived from the allocated physiological index (QFR or FFR). The primary endpoint of the trial is the level of agreement between the treatment recommendations of both teams, assessed using Cohen's *κ*. As of March 2022, the patient enrollment has been completed and 230 patients have been discussed in both Heart Teams. The current trial will indicate the usefulness of QFR, which enables a wireless multivessel physiological interrogation, in the discussions of Heart Teams to determine the optimal revascularization strategy for MVD.

## 1 INTRODUCTION

### 1.1 Background

An individualized approach for the management of patients with multivessel disease (MVD) requires that the optimal revascularization strategy (percutaneous coronary intervention [PCI] vs. coronary artery bypass graft [CABG]) be discussed by a multidisciplinary Heart Team. This discussion should consider the anatomical complexity of the coronary tree assessed using the SYNTAX score (SS).^1^, ^2^ The SS discriminates the prognostic risk in patients with MVD who require revascularization, and provides guidance on appropriate treatment recommendations.^3^

The SYNTAX score II (SSII) is a refined version of the SS and generates an estimate of 4‐year mortality. While SS considers only anatomical information, SSII also considers patient characteristics, such as age, sex, and comorbidities. Recently, SSII was redesigned (SYNTAX score II 2020) based on the extended follow‐up database in the SYNTAX trial, generating an estimate of the 10‐year mortality and 5‐year major adverse cardiac event (MACE) rate, which has been externally validated.^4^, ^5^

The original SS is calculated based on an angiographic diameter stenosis (DS) of 50%, with visual estimation. However, angiography‐guided PCI is associated with worse clinical outcomes compared with PCI guided by functional assessment using fractional flow reserve (FFR).^6^ Park et al.^7^ reported that visual‐functional mismatch was observed in 39.2% of non‐left‐main lesions (343/1066 with DS ≥ 50% and FFR > 0.80, 75/1066 with DS < 50% and FFR ≤ 0.80).This suggested that anatomical stenosis assessment did not always reflect the functional significance of lesions and therefore had limited capability to estimate the risk of the lesions.

The functional SYNTAX score (FSS) was developed as another approach to improve the prognostic capability of SS. In FSS, only lesions whose functional significance is confirmed using FFR are scored^8^; thus, a better risk stratification is achieved.^8^ Nevertheless, an FFR measurement requires an invasive procedure that can potentially lead to complications (e.g., coronary dissection) and patient discomfort.^9^

### 1.2 Risk stratification of MVD patients using angiography‐derived FFR

To date, various types of angiography‐derived FFR, such as Quantitative flow ratio (QFR®, Medis Medical Imaging), FFRangio® (Cathworks), and vFFR® (Pie Medical Imaging), have emerged and are commercially available with relatively simple mathematical formulas. These indices can be calculated based on three‐dimensional angiography in a short time without the need for a pressure wire or pharmacological hyperemia.^10^, ^11^, ^12^ QFR is the first CE‐marked angiography‐derived FFR and has been well investigated regarding not only the diagnostic performance but also its impact on clinical outcomes.^13^ It was reported that QFR‐guided PCI improved 1‐year clinical outcomes as compared with angiography‐guided PCI.^14^ The QFR‐derived functional SYNTAX score (FSS_QFR_) is an FSS calculated based on functional assessment using QFR.^15^, ^16^ Thus, it eliminates the risk of FFR measurement‐related complications and potentially reduces the procedure time for multivessel evaluations. The FSS_QFR_ demonstrated good reclassification from anatomical SS for predicting 2‐year patient‐oriented cardiovascular events (net reclassification improvement, 0.32; *p* < .001) in a post‐hoc study of the SYNTAX II trial.^15^ However, in that retrospective study, the FSS_QFR_ could be calculated in only 35.8% of patients because of the lack of two paired projections that met the requirements for QFR calculation.^15^ Accurate QFR calculation entails a precise three‐dimensional reconstruction of the coronary artery. This reconstruction in turn requires two adequate coronary angiographic projections (with angles ≥25° apart) including target lesions that are clearly visualized without vessel shortening or overlapping.^10^ For these requirements, prospective angiography considering QFR analysis is of paramount importance.

### 1.3 Impact of functional information on Heart Team discussion and the rationale of the current study

Compared with anatomical information alone, the addition of coronary functional information leads to better treatment decisions by the Heart Team. The SYNTAX III trial investigated the agreement of treatment decisions between a Heart Team using the angiography approach and that of a team using the coronary computed tomography angiography (CCTA) approach.^17^ The functional information based on the FSS and SSII using FFR_CT_ changed the Heart Team's treatment decision in 7% of patients and modified vessel selection for revascularization in 12% of patients.^18^

However, to the best of our knowledge, the feasibility of using QFR as the basis for treatment decision‐making in Heart Team discussions for patients with MVD has not been investigated. Thus, this prospective study aimed to evaluate the feasibility of using QFR‐based patient information based on the FSS_QFR_ and the FSS_QFR_‐based SSII (SSII_QFR_) in Heart Team discussions for the treatment of patients with MVD. The primary hypothesis of this study is that there is an acceptable agreement between the treatment recommendation based on the QFR approach and the recommendation based on the FFR approach.

## 2 METHODS

### 2.1 Study design

**Figure 1:**
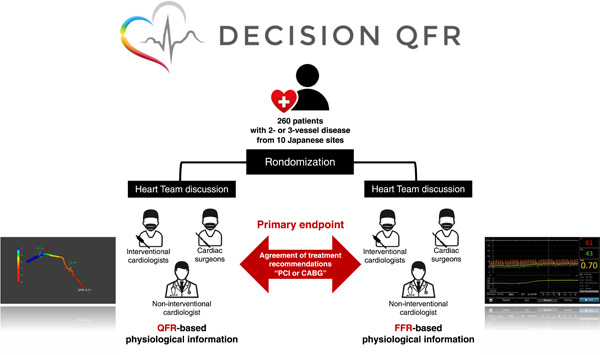
Trial design of the DECISION QFR study. The DECISION QFR trial is a multicenter, randomized controlled trial investigating the feasibility of quantitative flow ratio (QFR)‐based patient information for determining the optimal revascularization strategy during Heart Team discussions. We will assess the agreement between the treatment recommendations based on QFR and those based on fractional flow reserve (FFR). The primary endpoint is the agreement between the treatment options recommended based on the QFR and FFR approach, as assessed with Cohen's *κ*. CABG, coronary artery bypass graft; FFR, fractional flow reserve; PCI, percutaneous coronary intervention; QFR, quantitative flow ratio.

The DEtermination of the appropriate proCedure of revascularization In the multidisciplinary Heart Team discusSION based on Quantitative Flow Ratio (DECISION QFR) trial is a prospective, multicenter, single‐blinded randomized controlled trial that includes patients with MVD who require revascularization (PCI or CABG) (Figure 1). The trial will investigate the feasibility of QFR‐based patient information using FSS_QFR_ and SSII_QFR_ in virtual Heart Team discussions compared with FFR‐based patient information using FSS and SSII derived from wire‐based FFR (FSS_FFR_ and SSII_FFR_). We will evaluate the level of agreement between two treatment recommendations generated by two virtual Heart Teams that are randomized to either the “QFR approach” or the “FFR approach.” All patients will be assessed by both Heart Teams in a cross‐over design.

**Figure 2:**
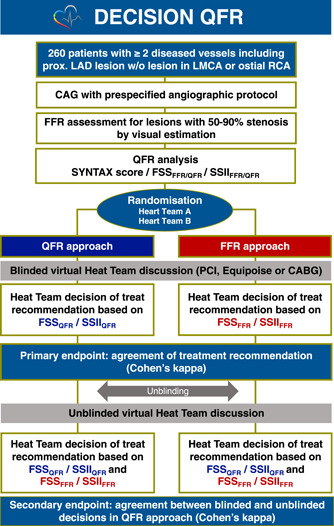
Study flowchart of the DECISION QFR trial. In the DECISION QFR study, two Heart Teams will be randomized to either patient information based on QFR or patient information based on FFR. Each team will have a virtual discussion and select a treatment recommendation for the revascularization strategy (percutaneous coronary intervention vs. coronary artery bypass graft). All 260 patients will be assessed by both teams with reference to the anatomical and functional SYNTAX score/SYNTAX score II 2020 derived from the allocated physiological index (QFR or FFR). FFR, fractional flow reserve; FSS, functional SYNTAX score; LAD, left anterior descending; LMCA, left main coronary artery; QFR, quantitative flow ratio; RCA, right coronary artery; SSII, SYNTAX score II.

In the QFR approach, the Heart Team makes treatment recommendations by referring to the patient information based on the FSS_QFR_/SSII_QFR_. In contrast, in the FFR approach, the Heart Team makes recommendations based on the FSS_FFR_/SSII_FFR_ (Figure 2). Randomization is performed using a web‐based randomizing module according to adaptive randomization. The teams are blinded to the allocation information. The level of agreement will be quantitatively evaluated using Cohen's *κ*. This trial design is inspired by the SYNTAX III trial.^17^

The research ethics committee of each participating institution has approved the protocol, and all enrolled patients have provided written informed consent before being included in the study. The current trial is registered at University Hospital Medical Information Network (UMIN000040475).

### 2.2 Inclusion and exclusion criteria

The major inclusion criteria of the current trial are as follows: (1) chronic coronary syndrome requiring revascularization (PCI or CABG); and (2) multiple lesions with DS of ≥50% (visual assessment) located in ≥2 vessels including the proximal left anterior descending (LAD; SYNTAX score segment: 6 and/or 7). The exclusion criteria are as follows: (1) left main coronary artery lesion or ostial lesion of right coronary artery (RCA) disease that is not recommended for the QFR analysis; (2) history of CABG; (3) advanced chronic kidney disease (estimated glomerular filtration rate <30 mL/min/1.73 m^2^) or receiving hemodialysis; (4) atrial fibrillation at the time of angiography; (5) severe valvular diseases; and (6) heart failure requiring oxygen supply (Table 1). Patients will be recruited from 10 Japanese sites listed in the Supporting Information.

**Table 1:** Major inclusion and exclusion criteria of the DECISION QFR trial

Inclusion criteria	Exclusion criteria
Chronic coronary syndrome requiring revascularization (PCI or CABG)	Left main coronary artery lesion or ostial lesion of RCA disease that is not recommended for the QFR analysis
Multiple lesions with %DS of ≥50% (visual assessment) located in ≥2 vessels including the proximal left anterior descending (LAD; SYNTAX score segment: 6 and/or 7)	History of CABG
	Advanced chronic kidney disease (estimated GFR < 30 ml/min/1.73 m^2^) or receiving hemodialysis
	Atrial fibrillation at the time of angiography
	Severe valvular diseases
	Heart failure requiring oxygen supply

Abbreviations: CABG, coronary artery bypass graft; DS, diameter stenosis; GFR, glomerular filtration rate; LAD, left anterior descending; PCI, percutaneous coronary intervention; QFR, quantitative coronary artery; RCA, right coronary artery.

### 2.3 Coronary angiography

Invasive coronary angiography (ICA) is performed as follows: (1) nitrate is administrated into the coronary arteries before ICA; (2) lesions need to be clearly visualized without vessel shortening or overlapping in at least two projections; (3) prespecified projection angles for QFR analysis (Table 2) are recommended; (4) cine angiography is recorded at ≥15 flames/s using radiographic systems; and (5) contrast medium is injected via a ≥5‐Fr catheter.

**Table 2:** Prespecified recommended projection angles

Right coronary artery	Left coronary arteries
LAO45°, CAUD10°	LAO30°, CRAN30°
LAO20°, CRAN20°	AP, CRAN45°
RAO30°, CAUD20°	RAO30°, CRAN20°
	RAO25°, CAUD25°
	RAO20°, CAUD45°
	AP, CAUD10°
	LAO10°, CAUD25°

Abbreviations: AP, anterior‐posterior; CAUD: caudal; CRAN: cranial; LAO, left anterior oblique; RAO, right anterior oblique.

### 2.4 FFR analysis

Lesions with ≥50% DS (with visual estimation) are assessed using FFR unless they have ≥90% DS. Pharmacologic hyperemia is induced using intravenous or intracoronary administration of adenosine triphosphate or intracoronary injection of papaverine or nicorandil. We will use a ≥5‐Fr guiding catheter and measure FFR at the distal part of the target lesions while ensuring not to reach a pressure sensor in the part of vessel with a diameter of <2.0 mm. For the co‐localization with QFR analysis, the positions of a pressure wire sensor are recorded on angiographic still images. The absence of major pressure signal drift (3 mmHg) is confirmed using a pullback maneuver after the FFR measurements. The procedure time during the FFR measurement (from zeroing a pressure wire to finishing all interrogations for entire lesions) will then be recorded.

### 2.5 QFR analysis

The lesions for which FFR is measured are eligible for the QFR analysis. Offline QFR analysis will be performed by an experienced analysts at an independent core laboratory (St. Luke's International Hospital, Tokyo, Japan) using the QAngio XA 3D software version 2.0 (Medis Medical Imaging Systems). One of the advantages of FSS based on QFR is that on‐site physiological assessment is not necessarily required because treatment decisions (i.e., Heart Team decision) are generally made after a patient leaves the catheter laboratory. Thus, we will apply the offline QFR analysis in the current trial. The analysts will be blinded to the FFR results. Details regarding the QFR calculation have been reported elsewhere.^10^ Briefly, the QFR calculation is based on the integration of segmental pressure gradients across stenotic lesions. These gradients are estimated using a mathematical formula that includes anatomical information derived from three‐dimensional quantitative coronary angiography reconstructed from two angiographic projections that are ≥25° apart and volumetric flow rate calculated using contrast bolus frame count.^10^ QFR is analyzed from the ostium of the main vessels (LAD, RCA, and left circumflex artery) to the anatomical site where the FFR is interrogated. The procedure time during QFR analysis (from opening cine files to finishing all analyses for entire lesions) is recorded.

### 2.6 Calculation of the SYNTAX score, SYNTAX score II 2020, and functional SYNTAX score

The SS, SSII, and FSS are calculated at the core laboratory using an app‐based calculator (https://syntaxscore2020.com). The SYNTAX II score 2020, the latest generation of the SSII, is applied for the SSII in the current trial. It generates two estimates of 5‐year MACE and 10‐year mortality rates for PCI and CABG treatments, respectively.^5^ The absolute difference in these prediction rates between PCI and CABG is used as reference in the Heart Teams' decision‐making. The FSSs are calculated based on both FFR and QFR assessment (FSS_FFR_ and FSS_QFR_) by summing the individual points of physiologically significant lesions (FFR and QFR ≤ 0.8) and excluding physiologically nonsignificant lesions.^15^ The SSIIs are calculated based on both anatomical SS and functional SSs derived from the FFR and QFR (SSII_FFR_ and SSII_QFR_).

### 2.7 Decision‐making of treatment recommendation in virtual Heart Teams

We created two Heart Teams (Heart Team A and Heart Team B) involving three cardiologists (two interventional cardiologists and one noninterventional cardiologist) and two cardiac surgeons from St. Luke's International Hospital. Each team will conduct virtual discussions and decides on the optimal treatment recommendation regarding the revascularization strategy (PCI or CABG). The strategy is decided according to the allocated functional information (FSS_QFR_/SSII_QFR_ or FSS_FFR_/SSII_FFR_) and the baseline patient information including cine angiography and anatomical SS and SSII. Each team decides a treatment recommendation from the following options:

(1) *CABG only*: The patient is recommended to receive CABG because the benefits of CABG are highlighted according to the risk assessment using (anatomical and functional) SS and SSII.
(2) *Equipoise*: The patient is recommended to receive either CABG or PCI because the benefits of each treatment are equipoised according to the risk assessment using (anatomical and functional) SS and SSII.
(3) *PCI only*: The patient is recommended to receive PCI because the benefits of PCI are highlighted according to the risk assessment using (anatomical and functional) SS and SSII.

The allocation information (QFR or FFR approach) is blinded before the first decision (primary endpoint). After the first decision, each team is provided with the allocation and opposite functional information (FSS_FFR_/SSII_FFR_ for QFR approach and FSS_QFR_/SSII_QFR_ for FFR approach), and the second decision is made based on identical information (unblinded decision).

### 2.8 Endpoints

The primary endpoint of the current trial is the level of agreement between the treatment recommendation based on the QFR approach and the recommendation based on the FFR approach. The level of agreement is assessed using Cohen's *κ* that will be calculated based on two decision components: CABG only and equipoise/PCI only.^17^ The secondary endpoints are as follows: (1) analyzability rate of FSS_QFR_ using a full set of analyzable QFR; (2) the level of agreement between target vessels for the revascularization (PCI and/or CABG) in the QFR approach and the target vessels in the FFR approach; (3) the level of agreement between decisions based on QFR information (blinded decision) and decisions after knowing the FFR information (unblinded decision) in the QFR approach; (4) the level of agreement between FSS_QFR_ and FSS_FFR_ assessed using interclass correlation coefficient (ICC); (5) procedure time of QFR analyses compared with that of FFR measurements; and (6) incidence rate of complications during FFR measurements.

### 2.9 Statistical considerations and sample size calculation

The level of agreement between the treatment recommendations will be assessed using Cohen's *κ* based on the decision components of “CABG only” and “equipoise/PCI only” as described above. A *κ* value of 0.61–0.80 between the two ratings is interpreted as a “substantial agreement,” and a *κ* value of 0.41–0.60 is considered a “moderate agreement.”^19^ We expect that the decision agreement will be substantial (*κ *> 0.60), while the trial will be considered positive if the lower boundary of the 95% confidence interval (CI) is >0.40. We expect that the kappa value will be lower than that in the *κ* value reached in the SYNTAX III trial (Cohen's *κ*: 0.82, 95% CI: 0.74–0.91) because the agreement of the treatment recommendations between the two teams will be deteriorated by using the SYNTAX score II 2020. The calculator of the original SYNTAX score II, which was applied in the SYNTAX III trial, offers treatment recommendations (i.e., “CABG only,” “CABG or PCI,” and “PCI only”) based on the specific thresholds of treatment effect of CABG over PCI in terms of 4‐year mortality.^4^ However, the calculator of the SYNTAX score II 2020 no longer offers treatment recommendations, and offers only an absolute difference of the predicted event rates (10‐year mortality and 5‐year MACE rates) between two treatment strategies. This is based on the idea that when one considers an individualized and patient‐centered care, it is difficult to determine the specific thresholds at which one treatment over the other should be initiated.^5^ This potentially leaves room for the interpretation of each Heart Team regarding the personalized benefit of a revascularization strategy for each patient. As a pilot study, we aim to test the agreement of treatment recommendations between Heart Team A and Heart Team B that share identical information (patients' baseline characteristics, anatomical SS, and SSII 2020 based on anatomical SS) for 21 patients with MVD. Cohen's *κ* for the treatment recommendation agreement was 0.63 [95% CI: 0.25–1.00], when 85.7% (18/21) agreement of the recommendation is observed. We will also evaluate the level of recommendation agreement between the two Heart Teams after unblinding to identify a between‐team decision variance.

We assume that both approaches will result in “CABG only” recommendation in 20% of the enrolled patients, with a *κ* of 0.60. According to the calculation with “irr” package in R version 3.6.2 (R Foundation), a sample size of 235 will be sufficient to achieve 80% power and reach a positive trial with a two‐sided *α* of 0.05. Assuming an attrition rate of 10%, to account for nonanalyzable cases, a total of 260 patients will be included in the current trial.

In the current study, we will exclude those patients without a full‐set QFR measurement from the primary analysis because values cannot be assigned to vessels without measuring the QFR. As a countermeasure for an inflated nonanalyzable rate, we recommend that operators should perform angiography with prospective consideration of QFR analysis or to use a prespecified angiographic protocol (Table 2).

## 3 DISCUSSION

Invasive functional assessment using a wire‐based functional index (e.g., FFR) is recommended for deciding on revascularization in patients with chronic coronary syndrome.^20^, ^21^ However, the global adoption rate of invasive functional assessment in catheter laboratories is still low.^22^ This may be because of the time‐consuming process of advancing the pressure wire beyond the target lesions and the high cost of the pressure wire and adenosine. Operators may also consider potential complications related to the use of a pressure wire and adenosine. As such, multivessel assessments of FFR are avoided in catheter laboratories despite the demonstrated clinical benefit of applying an FSS. Angiography‐derived FFR potentially increases the practicability of FSS without using a pressure wire or pharmacological hyperemia. Another advantage of QFR is that it can be analyzed in a post‐hoc manner, thus avoiding lengthy patient stays in the catheter laboratory. The current trial will investigate the comparability of QFR with wire‐based FFR in terms of the validity as a functional information in Heart Team discussion. This result will also push functional assessment in the Heart Team toward being more practical.

We set the primary endpoint of the current trial as an agreement between two Heart Teams' treatment decisions, which will be generated based on the functional and anatomical information representing FSS_FFR_ or FSS_QFR_ as well as the patients' baseline characteristics. We consider that QFR/FFR in the Heart Teams' discussions plays a significant role not only for the FSS but also for the per‐vessel functional information. FSS is a reasonable tool that can be used to summarize the anatomical complexity of patients while considering functional significance. However, as a consequence of summarization, the score itself does not present lesion information, such as the locations of functionally significant lesions, whereas the score is weighted based on the location of the lesion. For example, even with identical FSS points, one patient may have a functionally significant lesion(s) in the LAD while another may not have significant lesions in the LAD. This information is crucial for the decision‐making regarding the optimal revascularization strategy in the Heart Teams' discussion. We consider that the misclassification of QFR especially in the LAD potentially impacts the Heart Teams' decision even if the FSS_FFR_ and FSS_QFR_ are comparable. Furthermore, in the current trial, the Heart Teams will discuss optimal treatment strategy by considering the vessel‐by‐vessel feasibility of each revascularization mode based on the functional information and lesion characteristics (severe calcification, severe tortuosity, complex bifurcation lesion, etc.). Per‐vessel functional information will also play an important role in the Heart Teams' decisions.

In the current trial, the Heart Teams will use QFR/FFR as a surrogate marker of myocardial ischemia, in which severity is generally graded continuously, while the functional SYNTAX score will be calculated based on each physiological index as a dichotomized parameter (≤0.8 or >0.8) according to the historical definition. ^8^ In the Heart Teams' discussion, each physiological index will also be used by considering its limitation in terms of reproducibility as previously reported based on a continuous ischemic spectrum.^23^, ^24^

FFR_CT_® (Heart Flow) is another FFR simulation and a potential tool for FSS calculation that does not require the use of a pressure wire.^17^ FFR_CT_ enables a noninvasive anatomical and functional assessment of a coronary tree without requiring ICA. Thus, from the viewpoint of invasiveness, FFR_CT_ is more favorable compared with QFR. Furthermore, this technology implemented a sophisticated computed fluid dynamics model based on Navier−Stokes equations that separately considers the inlet and outlet flows among coronary artery branches while QFR calculation is based on Hagen‐Poiseuille and Borda‐Carnot equations; this simplifies the model by considering that the coronary artery is a single straight tubular model while using the volumetric flow estimated from contrast frame count.^10^, ^25^ However, it has been reported that the diagnostic performance of QFR with FFR as reference (area under the curve 0.93 [95% CI: 0.89−0.95]) is superior to that of FFR_CT_ (0.82 [95% CI: 0.76−0.87]).^26^ Compared with FFR_CT_, QFR may have an advantage of better diagnostic accuracy as it generates an FSS with better prognostic value. Nevertheless, it should be appreciated that the simple model used in QFR calculation potentially yields reduced accuracy in complex lesions which are often observed in patients with MVD. In the substudy of the SYNTAX II trial enrolling patients with three‐vessel disease, deteriorated diagnostic performance was reported for bifurcation lesions and small vessels.^15^ The SYNTAX III trial investigated the additional impact of the addition of FFR_CT_ to anatomical information (obtained via CCTA) on the Heart Team decision‐making as described above. Nevertheless, the trial neither mandated FFR measurements nor investigated the usefulness of FFR_CT_‐based patient risk assessment in comparison with that of a conventional functional assessment using FFR. The current trial will compare the feasibility of QFR‐based patient risk assessment as an alternative to FFR‐based patient risk assessment in the decision‐making of Heart Teams.

Recently, several studies revisited the role of physiology‐guided revascularization in patients with MVD. In the post‐hoc analysis of the ISCHEMIA trial, which was presented at the annual scientific session of American College of Cardiology in 2021, anatomical complete revascularization was found to be superior to conservative management in terms of long‐term composite cardiovascular endpoints (11.8% vs. 15.4% adjusted difference −3.6% [−6.9 to −0.7%]) whereas functional complete revascularization was not found to be superior to conservative management (12.9% vs. 15.4% adjusted difference −2.5% [−5.7% to 0.3%]). Although these findings were not supported with adequate sample size and just represented associations and not causality, it was suggested that anatomically complete revascularization may be more important than functionally complete revascularization. Furthermore, the FUTURE trial did not prove the advantages of FFR‐guided treatment strategy in terms of 1‐year ischemic cardiovascular events or death compared with angiography‐guided revascularization strategy.^27^ The trial enrolled multivessel disease patients in whom CABG was also one of the treatment options. The FAME trial also investigated and successfully proved the efficacy of FFR‐guided PCI in patients with MVD.^6^ However, this trial included only candidates for PCI treatment and not those with disease severe enough for CABG treatment. Indeed, the FUTURE trial enrolled patients with more severe lesions in terms of anatomical complexity and patient age than the FAME trial. In patients with more severe lesions and high atherosclerotic burden, FFR may not always be useful. Extensive atherosclerosis leads to microvascular dysfunction, and in such cases, both FFR and QFR have decreased diagnostic performance.^28^, ^29^, ^30^ Furthermore, when CABG is being considered as a treatment strategy, the role of the physiological indices should be discussed separately. ^31^ In our trial, we will discuss these points based on the upcoming results.

## 4 CONCLUSION

The DECISION QFR trial will be the first prospective study to investigate the feasibility of QFR using FSS_QFR_ and SSII_QFR_ in Heart Team discussions for the risk assessment of patients with MVD. QFR is a potentially useful tool for multivessel functional assessment owing to its advantage of being minimally invasive.

## CONFLICTS OF INTEREST

Prof. Reiber is the CSO of Medis Medical Imaging Systems and has a part‐time appointment at Leiden University Medical Center as a professor of medical imaging. All other authors have no conflict of interest to disclose. The authors declare no conflicts of interest.

## Supporting information

Supplementary information.

## Data Availability

Data sharing not applicable—no new data generated.

## References

[1] Neumann FJ , Sousa‐Uva M , Ahlsson A , et al. ESC/EACTS Guidelines on myocardial revascularization. Eur Heart J. 2018;40(2):87‐165. 10.1093/eurheartj/ehy855 30615155

[2] Patel MR , Calhoon JH , Dehmer GJ , et al. ACC/AATS/AHA/ASE/ASNC/SCAI/SCCT/STS 2017 Appropriate use criteria for coronary revascularization in patients with stable ischemic heart disease: a Report of the American College of Cardiology Appropriate Use Criteria Task Force, American Association for Thoracic Surgery, American Heart Association, American Society of Echocardiography, American Society of Nuclear Cardiology, Society for Cardiovascular Angiography and Interventions, Society of Cardiovascular Computed Tomography, and Society of Thoracic Surgeons. J Am Coll Cardiol. 2017;69(17):2212‐2241. 28291663 10.1016/j.jacc.2017.02.001

[3] Serruys PW , Morice MC , Kappetein AP , et al. Percutaneous coronary intervention versus coronary‐artery bypass grafting for severe coronary artery disease. N Engl J Med. 2009;360(10):961‐972. 19228612 10.1056/NEJMoa0804626

[4] Farooq V , van Klaveren D , Steyerberg EW , et al. Anatomical and clinical characteristics to guide decision making between coronary artery bypass surgery and percutaneous coronary intervention for individual patients: development and validation of SYNTAX score II. Lancet. 2013;381(9867):639‐650. 23439103 10.1016/S0140-6736(13)60108-7

[5] Takahashi K , Serruys PW , Fuster V , et al. Redevelopment and validation of the SYNTAX score II to individualise decision making between percutaneous and surgical revascularisation in patients with complex coronary artery disease: secondary analysis of the multicentre randomised controlled SYNTAXES trial with external cohort validation. Lancet. 2020;396(10260):1399‐1412. 33038944 10.1016/S0140-6736(20)32114-0

[6] Tonino PA , De Bruyne B , Pijls NH , et al. Fractional flow reserve versus angiography for guiding percutaneous coronary intervention. N Engl J Med. 2009;360(3):213‐224. 19144937 10.1056/NEJMoa0807611

[7] Park SJ , Kang SJ , Ahn JM , et al. Visual‐functional mismatch between coronary angiography and fractional flow reserve. JACC Cardiovasc Interv. 2012;5(10):1029‐1036. 23078732 10.1016/j.jcin.2012.07.007

[8] Nam CW , Mangiacapra F , Entjes R , et al. Functional SYNTAX score for risk assessment in multivessel coronary artery disease. J Am Coll Cardiol. 2011;58(12):1211‐1218. 21903052 10.1016/j.jacc.2011.06.020

[9] Kern MJ , Donohue TJ , Aguirre FV , et al. Clinical outcome of deferring angioplasty in patients with normal translesional pressure‐flow velocity measurements. J Am Coll Cardiol. 1995;25(1):178‐187. 7798498 10.1016/0735-1097(94)00328-n

[10] Tu S , Westra J , Yang J , et al. Diagnostic accuracy of fast computational approaches to derive fractional flow reserve from diagnostic coronary angiography: the International Multicenter FAVOR Pilot Study. JACC Cardiovasc Interv. 2016;9(19):2024‐2035. 27712739 10.1016/j.jcin.2016.07.013

[11] Fearon WF , Achenbach S , Engstrom T , et al. Accuracy of fractional flow reserve derived from coronary angiography. Circulation. 2019;139(4):477‐484. 30586699 10.1161/CIRCULATIONAHA.118.037350

[12] Masdjedi K , Tanaka N , Van Belle E , et al. Vessel fractional flow reserve (vFFR) for the assessment of stenosis severity: the FAST II study. *EuroIntervention*. Published online October 14, 2021. 10.4244/EIJ-D-21-00471 PMC9896401 34647890

[13] Westra J , Tu S , Campo G , et al. Diagnostic performance of quantitative flow ratio in prospectively enrolled patients: an individual patient‐data meta‐analysis. Catheter Cardiovasc Interv. 2019;94(5):693‐701. 30963676 10.1002/ccd.28283

[14] Xu B , Tu S , Song L , et al. Angiographic quantitative flow ratio‐guided coronary intervention (FAVOR III China): a multicentre, randomised, sham‐controlled trial. Lancet. 2021;398(10317):2149‐2159. 34742368 10.1016/S0140-6736(21)02248-0

[15] Asano T , Katagiri Y , Chang CC , et al. Angiography‐derived fractional flow reserve in the SYNTAX II trial: feasibility, diagnostic performance of quantitative flow ratio, and clinical prognostic value of functional SYNTAX score derived from quantitative flow ratio in patients with 3‐vessel disease. JACC Cardiovasc Interv. 2019;12(3):259‐270. 30409759 10.1016/j.jcin.2018.09.023

[16] Zhang R , Song C , Guan C , et al. Prognostic value of quantitative flow ratio based functional SYNTAX score in patients with left main or multivessel. coronary artery disease. Circ Cardiovasc Interv. 2020;13(10):e009155. 33040580 10.1161/CIRCINTERVENTIONS.120.009155

[17] Collet C , Onuma Y , Andreini D , et al. Coronary computed tomography angiography for heart team decision‐making in multivessel coronary artery disease. Eur Heart J. 2018;39(41):3689‐3698. 30312411 10.1093/eurheartj/ehy581 PMC6241466

[18] Andreini D , Modolo R , Katagiri Y , et al. Impact of fractional flow reserve derived from coronary computed tomography angiography on heart team treatment decision‐making in patients with multivessel coronary artery disease: insights from the SYNTAX III REVOLUTION trial. Circ Cardiovasc Interv. 2019;12(12):e007607. 31833413 10.1161/CIRCINTERVENTIONS.118.007607

[19] Landis JR , Koch GG . The measurement of observer agreement for categorical data. Biometrics. 1977;33(1):159‐174. 843571

[20] Knuuti J , Wijns W , Saraste A , et al. ESC Guidelines for the diagnosis and management of chronic coronary syndromes. Eur Heart J. 2019;41(3):407‐477. 10.1093/eurheartj/ehz425 31504439

[21] Fihn SD , Gardin JM , Abrams J , et al. ACCF/AHA/ACP/AATS/PCNA/SCAI/STS Guideline for the diagnosis and management of patients with stable ischemic heart disease: a report of the American College of Cardiology Foundation/American Heart Association Task Force on Practice Guidelines, and the American College of Physicians, American Association for Thoracic Surgery, Preventive Cardiovascular Nurses Association, Society for Cardiovascular Angiography and Interventions, and Society of Thoracic Surgeons. J Am Coll Cardiol. 2012. 2012;60(24):e44‐e164. 10.1016/j.jacc.2012.07.013 23182125

[22] Gotberg M , Cook CM , Sen S , Nijjer S , Escaned J , Davies JE . The evolving future of instantaneous wave‐free ratio and fractional flow reserve. J Am Coll Cardiol. 2017;70(11):1379‐1402. 28882237 10.1016/j.jacc.2017.07.770

[23] Petraco R , Sen S , Nijjer S , et al. Fractional flow reserve‐guided revascularization: practical implications of a diagnostic gray zone and measurement variability on clinical decisions. JACC Cardiovasc Interv. 2013;6(3):222‐225. 23517831 10.1016/j.jcin.2012.10.014

[24] Westra J , Sejr‐Hansen M , Koltowski L , et al. Reproducibility of quantitative flow ratio: the QREP study. EuroIntervention. 2022;17(15):1252‐1259. 34219667 10.4244/EIJ-D-21-00425 PMC9724855

[25] Giddens DP . Computing fractional flow reserve during coronary angiography: how good is good enough? JACC Cardiovasc Interv. 2016;9(19):2036‐2038. 27712740 10.1016/j.jcin.2016.08.003

[26] Tanigaki T , Emori H , Kawase Y , et al. QFR versus FFR derived from computed tomography for functional assessment of coronary artery stenosis. JACC Cardiovasc Interv. 2019;12(20):2050‐2059. 31648766 10.1016/j.jcin.2019.06.043

[27] Rioufol G , Dérimay F , Roubille F , et al. Fractional flow reserve to guide treatment of patients with multivessel coronary artery disease. J Am Coll Cardiol. 2021;78(19):1875‐1885. 34736563 10.1016/j.jacc.2021.08.061

[28] Meuwissen M , Chamuleau SAJ , Siebes M , et al. Role of variability in microvascular resistance on fractional flow reserve and coronary blood flow velocity reserve in intermediate coronary lesions. Circulation. 2001;103(2):184‐187. 11208673 10.1161/01.cir.103.2.184

[29] Mejía‐Rentería H , Lee JM , Lauri F , et al. Influence of microcirculatory dysfunction on angiography‐based functional assessment of coronary stenoses. JACC Cardiovasc Interv. 2018;11(8):741‐753. 29673505 10.1016/j.jcin.2018.02.014

[30] Gurunathan S , Ahmed A , Vamvakidou A , et al. Diagnostic concordance and clinical outcomes in patients undergoing fractional flow reserve and stress echocardiography for the assessment of coronary stenosis of intermediate severity. J Am Soc Echocardiogr. 2018;31(2):180‐186. 29246509 10.1016/j.echo.2017.10.012

[31] Thuesen AL , Riber LP , Veien KT , et al. Fractional flow reserve versus angiographically‐guided coronary artery bypass grafting. J Am Coll Cardiol. 2018;72(22):2732‐2743. 30497559 10.1016/j.jacc.2018.09.043

