# Supplementary material for: Heart Team risk assessment with angiography‐derived fractional flow reserve determining the optimal revascularization strategy in patients with multivessel disease: Trial design and rationale for the DECISION QFR randomized trial
Source: Clin Cardiol. 2022 Mar 31;45(6):605–13. doi: 10.1002/clc.23821 (PMC9175249; doi:10.1002/clc.23821)
Supplement: Supplementary file 1 — Supplementary information. [file CLC-45-605-s001.docx]

**Supplementary appendix**

**List of participating hospitals**

Sapporo Higashi Tokushukai Hospital, Sapporo, Japan

Tsuchiura Kyodo General Hospital, Ibaraki, Japan

Mitsui Memorial Hospital, Tokyo, Japan

St. Luke’s International Hospital, Tokyo, Japan

Toho University Ohashi Medical Center, Japan

Gifu Heart Centre, Gifu, Japan

Osaka Police Hospital, Osaka, Japan

Wakayama Medical University, Wakayama, Japan

Yamaguchi University, Yamaguchi, Japan

Kokura Memorial Hospital, Kitakyushu, Japan
